# Dermoscopic findings in a case of plasma cell cheilitis^☆^

**DOI:** 10.1016/j.abd.2020.12.019

**Published:** 2022-09-08

**Authors:** Daniella Truffello, Carolina Cevallos, Claudio Escanilla, Pauline Morgan

**Affiliations:** a Dermatology Resident, Universidad de Santiago de Chile, Santiago, Chile; b Dermatology Department, Hospital Barros Luco Trudeau, Santiago, Chile; c Pathology Department, Hospital Regional Libertador Bernardo O’Higgins, Rancagua, Chile

Dear Editor,

Plasma Cell Cheilitis (PCC) is a rare inflammatory disorder of unknown origin within the spectrum of plasma cell mucositis. Clinically, it manifests as a circumscribed, flat to slightly raised, eroded erythematous plaque or patch involving the lower lip of elderly male patients.^1^ Histopathologically, dense band-like plasma cell infiltration in the upper dermis is seen.^2^ Dermoscopic features of this entity have been described in only one report.^3^ Here we report a case of refractory PCC and its dermoscopic features.

An otherwise healthy 52-year-old man, an agricultural worker, was referred to our hospital with a ten-year history of painful erythematous erosion on the lower lip. Physical examination revealed an erythematous plaque with diffuse desquamation along with erosions and crusts (Fig. 1A). Dermoscopy showed a well-defined lesion with the milky-red structureless area, small erosions, and multiple enlarged linear vessels on the periphery with a radial distribution. Scales although present on a small focus of the lesion was not a predominant feature (Fig. 2). Laboratory tests, including complete blood counts and tests for liver and renal function, showed normal findings, and hepatitis B and C and HIV infection was negative; PPD; chest X-Ray; thyroid tests and protein electrophoresis were in normal ranges. Histopathology showed a partially ulcerated pluriestratified epithelium with parakeratosis, without atypia, with pseudoepitheliomatous hyperplasia (Fig. 3A). The dermis showed foci of chronic inflammatory infiltrate with abundant plasma cells (Fig. 3B), macrophages, and areas of granulation tissue. The immuno-histochemical study revealed CK AE1/AE3 (-), CD68 (+) in membrane and macrophage cytoplasm, staining positive Kappa (Fig. 3C) and Lambda chains (Fig. 3D). The patient began treatment with high potency topical and intralesional corticosteroids, with little response. After the use of oral prednisone at a dose of 1 mg/kg/day, he presented total remission of the lesion on the seventh day (Fig. 1B). However, the lesion constantly recurs during tapering. Treatment with topical calcineurin inhibitors was not possible due to economic concerns.

**Figure 1:**
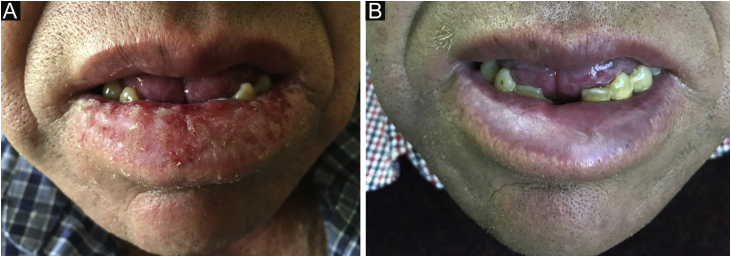
(A) The lower lip shows a diffuse xerotic erythematous plaque with erosions and hemorrhagic crusts. (B) Clinical improvement after 7-days of treatment with oral prednisone.

**Figure 2:**
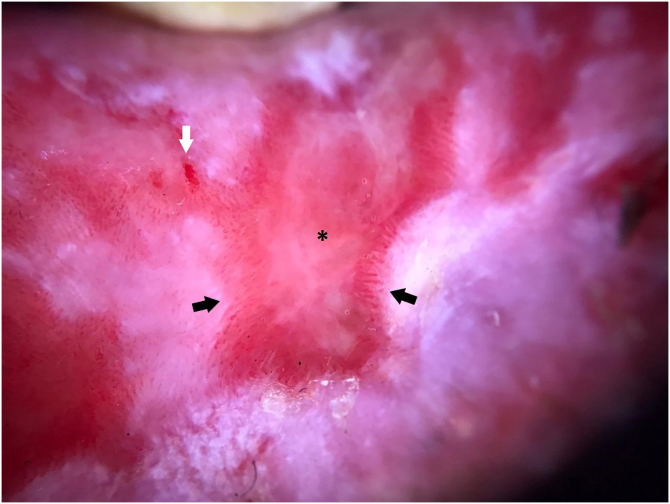
Dermoscopy of PCC shows a sharply demarcated borders with milky-red structureless area (asterisk), small erosions (white arrow), and multiple enlarged linear vessels on the periphery with a radial distribution (black arrow); a small focus of white scale on the inferior aspect of the lesion is also seen.

**Figure 3:**
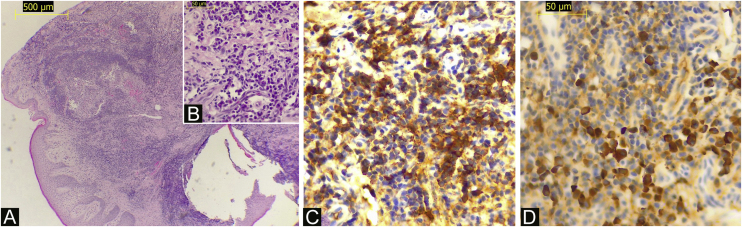
(A) A biopsy specimen shows a flattened squamous epithelium with parakeratosis without atypia, with pseudoepitheliomatous hyperplasia (Hematoxylin & eosin, ×100). (B) A high-power view shows a dermal chronic inflammatory infiltrate with abundant plasma cells (Hematoxylin & eosin, ×400). (C) A diffuse strong positivity for kappa light chain is noted in the plasma cell infiltrates (Immunohistochemistry, ×400). (D) Plasma cells are also positive for lambda light chain (Immunohistochemistry, ×400).

One of the main differential diagnoses in the context of our patient was Actinic Cheilitis (AC) or even progression to Squamous Cell Carcinoma (SCC). Dermoscopy could be a useful tool to help differentiate these entities. We found some similarities between our case and the previously reported regarding border regularity and vascular enlargement and proliferation.^3^ Other important characteristics found in this case were the milky-red background in the entire lesion with some focal erosions, the absence of stellate border and scales (features suggestive of AC), and radial distribution of the enlarged lineal vessels on the periphery. More reports are needed to establish clear-cut criteria to aid in the clinical differentiation of these entities.

Although PCC is considered a benign disorder is usually refractory to various topical treatments, including topical and intralesional corticosteroids, topical calcineurin inhibitors, antibiotics, and antifungal agents.^1^, ^2^ It is unclear whether PCC may represent a precursor lesion of malignancies such as SCC.^4^ Therefore, careful follow-up is recommended.

## Financial support

None declared.

## Authors’ contributions

Daniella Truffello: Approval of the final version of the manuscript; critical literature review; data collection, analysis, and interpretation; intellectual participation in propaedeutic and/or therapeutic management of studied cases; study conception and planning.

Carolina Cevallos: Approval of the final version of the manuscript; critical literature review; effective participation in research orientation; manuscript critical review; preparation and writing of the manuscript; study conception and planning.

Claudio Escanilla: Approval of the final version of the manuscript; critical literature review; effective participation in research orientation; intellectual participation in propaedeutic and/or therapeutic management of studied cases; manuscript critical review; study conception and planning.

Pauline Morgan: Approval of the final version of the manuscript; data collection, analysis and interpretation; intellectual participation in propaedeutic and/or therapeutic management of studied cases.

## Conflicts of interest

None declared.
